# Cincho-Quinine

**Published:** 1875-10

**Authors:** 


*Cincho-Quinine.*

The attention of the medical profession has frequently been called to an agent which, in many respects, we believe to be superior to sulphate of quinine as a remedy for malarious fevers.

It is well known that the Madras Government appointed a commission to test the respective efficacy of the various alkaloids of Peruvian bark in the treatment of fever, and from their report it appears that the number of cases of paroxysmal malarious fever treated was 2,472.

The difference in the remedial value of the four alkaloids, as deduced from these statements, may be thus stated:

Quinidine, ratio of failure per 1,000 cases............ 6
Qunine, ratio of failure per 1,000 cases........... .. 7
Cinchonidine, ratio of failure per 1,000 cases.........10
Cinchonine, ratio of failure per 1,000 cases...........23

The Indian Government, acting on their report, officially advised (December 16, 1873,) the more free use in India of cinchonia alkaloids other than quinine.

In another report of the same commission we find this statement:

"In small doses all the alkaloids produce the same therapeutic effects; that is, as tonics, anti-periodics, and anti-neuralgics. Hypodermically and internally, they have proved successful. In large doses they are all equally energetic, and produce their effects rapidly. They have been successfully used as anti-periodics and febrifuges."

Cincho-quinine is valuable to the profession, from the fact that in it are combined the alkaloidal principles of bark, quinia, quinidia, cinchonidia, cinchonia, and the testimony of those who have prescribed it (extensively) is, that these alkaloids, acting in association, produce more favorable remedial influence than any one alone.

Furthermore, while being nearly tasteless, it exerts the full therapeutic effect of the sulphate of quinine, in the same doses, without oppressing the stomach, creating nausea, or producing cerebral distress.

In a former number of the RECORD, you published the results of the chemical analyses of the agent by distinguished chemists, and we prefer to accept the statements of such men as Professor F. A. Genth, of the University of Pennsylvania; Professor C. Gilbert Wheeler, of the University of Chicago; Professor C. F. Chandler, of Columbia College, New York, to any analysis by pharmacists who differ so widely in the results of their experiments, as is shown in the following letter, which is published in the May number of the *American Journal of Pharmacy.* C. E. D.

"BOSTON, April 15, 1875.

"*Editor American Journal of Pharmacy:*

"DEAR SIR—Our attention having been called to a communication by Messrs. E. Scheffer and C. L. Diehl, in the April number of the *American Journal of Pharmacy,* purporting to be a chemical examination of cincho-quinine, we desire to remark briefly as follows:

"The agent was introduced to the profession in 1869, since which no change whatever has been made in its composition. During this period it has been examined by four pharmacists: First, by Mr. W. T. Wenzell, of San Francisco, in 1870. Second, by A. E. Ebert, of Chicago, in 1874; and lastly, by Messrs. E. Scheffer and C. L. Diehl, of Louisville.

"The result of Mr. Wenzell's analysis was the discovery of two substances or principles which the agent did not contain, and he failed to discover quinia, quinidia or cinchonidia. Mr. Ebert was able to discover only cinchonia, failing utterly to find quinia, quinidia or cinchonidia. Messrs. Scheffer and Diehl find quinia, quinidia and cinchonia; and they remark (page 159) that, 'if it contains cinchonidia, it can be present only in small quantities, and they did not search for it.'"

"The widely different conclusions reached in the qualitative examinations made by these gentlemen must lead the reader to conclude, with us, that when an agent is made up of such complex and delicate organic principles as are found in barks, and the tests and reactions involve deceptive color-tints or forms of crystals with such varying solubility, and when these tests are so frequently fallacious and unreliable, the pharmacist and the chemist should be careful in expressing positive opinion respecting the results of their investigations when they differ from the statements of the manufacturer, who certainly has the most reliable knowledge upon the subject.

"As manufacturers of the article, we unhesitatingly say that never has a phial of the agent left our laboratory constituted in correspondence with the quantitative results reached by the Louisville gentlemen, and, in saying this, we distinctly disclaim any purpose of charging them with intentional false statements.

"In our circulars we have stated cincho-quinine to be the bark alkaloids quinia, cinchonia, quinidia, cinchonidia, and other alkaloidal principles present in Peruvian barks, and it contains no substances but those naturally existing in bark. By this, we wish to be understood as stating that cincho-quinine is a new method of presenting the bark alkaloids, and is unlike any other product which may be substituted by physicians for sulphate of quinine.

"In conclusion, we are convinced that no more certain proof of the remedial value of cincho-quinine could be given than its growing popularity with the medical profession, and the attention given to it by the pharmacists of the country; and while their published analyses, both qualitative and quantitative, differ widely from each other, and all are incorrect, it is yet a source of satisfaction to us that the more exhaustive their labors, the more nearly do they approach to our statements regarding its nature and value.

"BILLINGS, CLAPP & CO."

We publish the above articles from two of our most esteemed friends on cincho-quinine, with the hope that much good will be accomplished by calling the attention of the profession to the different opinions as to the real elements and therapeutical effects of this comparatively new and popular agent. P.

